# Exosomes and Metabolic Function in Mice Exposed to Alternating Dark-Light Cycles Mimicking Night Shift Work Schedules

**DOI:** 10.3389/fphys.2017.00882

**Published:** 2017-11-02

**Authors:** Abdelnaby Khalyfa, Valeriy A. Poroyko, Zhuanhong Qiao, Alex Gileles-Hillel, Ahamed A. Khalyfa, Mahzad Akbarpour, Isaac Almendros, Ramon Farré, David Gozal

**Affiliations:** ^1^Section of Pediatric Sleep Medicine, Department of Pediatrics, Pritzker School of Medicine, Biological Sciences Division, University of Chicago, Chicago, IL, United States; ^2^Unitat de Biofísica i Bioenginyeria, Facultat de Medicina i Ciències de la Salut, Universitat de Barcelona, Barcelona, Spain; ^3^CIBER de Enfermedades Respiratorias, Madrid, Spain; ^4^Institut d'investigacions Biomèdiques August Pi i Sunyer, Barcelona, Spain

**Keywords:** shift work, exosomes, insulin resistance, microbiota and immunity, macrophage polarity, clock gene

## Abstract

Sleep is an important modulator of metabolic function. Disruptions of sleep in circadian rhythm are common in modern societies and are associated with increased risk of developing cardiometabolic disorders. Exosomes are ubiquitous extracellular vesicles that may play a mechanistic role in metabolic derangements. We hypothesized that alternating dark-light cycles mimicking shift work in mice would alter fecal microbiota and colonic epithelium permeability and alter plasma exosome cargo and metabolic function. C57BL/6 mice were randomly assigned to (i) control day light (CL), or (ii) inverted dark-light every 2 weeks for 8 weeks (IN). Body weight, fat mass and HOMA-IR were measured, along with Tregs, metabolic, and resident macrophages in visceral white adipose tissue (vWAT). Fecal water samples were incubated with confluent colonic epithelium cell cultures in electric cell-substrate impedance sensing (ECIS) arrays, and plasma exosomes were added to differentiated adipocytes and insulin-induced pAKT/AKT expression changes were assessed by western blots. Mice exposed to IN showed elevated HOMA-IR, and their fecal samples showed altered microbiota which promote increased permeability of the colonic epithelial cell barrier. Plasma exosomes decreased pAKT/AKT responses to exogenous insulin compared to CL, and altered expression of circadian clock genes. Inflammatory macrophages (Ly-6c^high^) were increased in IN-exposed vWAT, while Tregs were decreased. Thus, gut microbiota and the cargo of plasma exosomes are altered by periodic shifts in environmental lighting, and effectively alter metabolic function, possibly via induction of systemic inflammation and altered clock expression in target tissues. Further exploration of exosomal miRNA signatures in shift workers and their putative metabolic organ cell targets appears warranted.

## Introduction

Sleep disturbance has been linked to adverse health outcomes including cardiovascular disease, obesity, diabetes, depression, and anxiety, as well as safety issues related to drowsy driving and injuries (Kim et al., 2015; Shockey and Wheaton, 2017). By disrupting circadian rhythms, shift work has emerged as an important risk factor of many health outcomes (Gumenyuk et al., 2012; Wyse et al., 2017). Nocturnal shiftwork alters the rhythmicity of the immune system, and such a disruption might play a role in the increased infection risk and higher incidence of autoimmune diseases, cancer, and cardiovascular and metabolic disorders (Esquirol et al., 2011). It has been suggested that the increase in artificial light exposure in modern societies parallels the increase in obesity prevalence, with altered circadian homeostasis clearly contributing to cardiovascular and metabolic derangements (Versteeg et al., 2016; Kiehn et al., 2017; Mayeuf-Louchart et al., 2017; Steffens et al., 2017; Tarquini and Mazzoccoli, 2017). Shift work, especially night work and long working hours are among the possible modifiable risk factors that influence the incidence of cardiovascular and metabolic risk factors (Puttonen et al., 2010; Nabe-Nielsen et al., 2017) and sleep (Sallinen and Kecklund, 2010; McHill et al., 2014; Givens et al., 2015; Wirth et al., 2017; Wyse et al., 2017). However, the molecular mechanisms linking insufficient sleep, chronic circadian perturbations, and insulin resistance remain largely unexplored.

**Figure 1:**
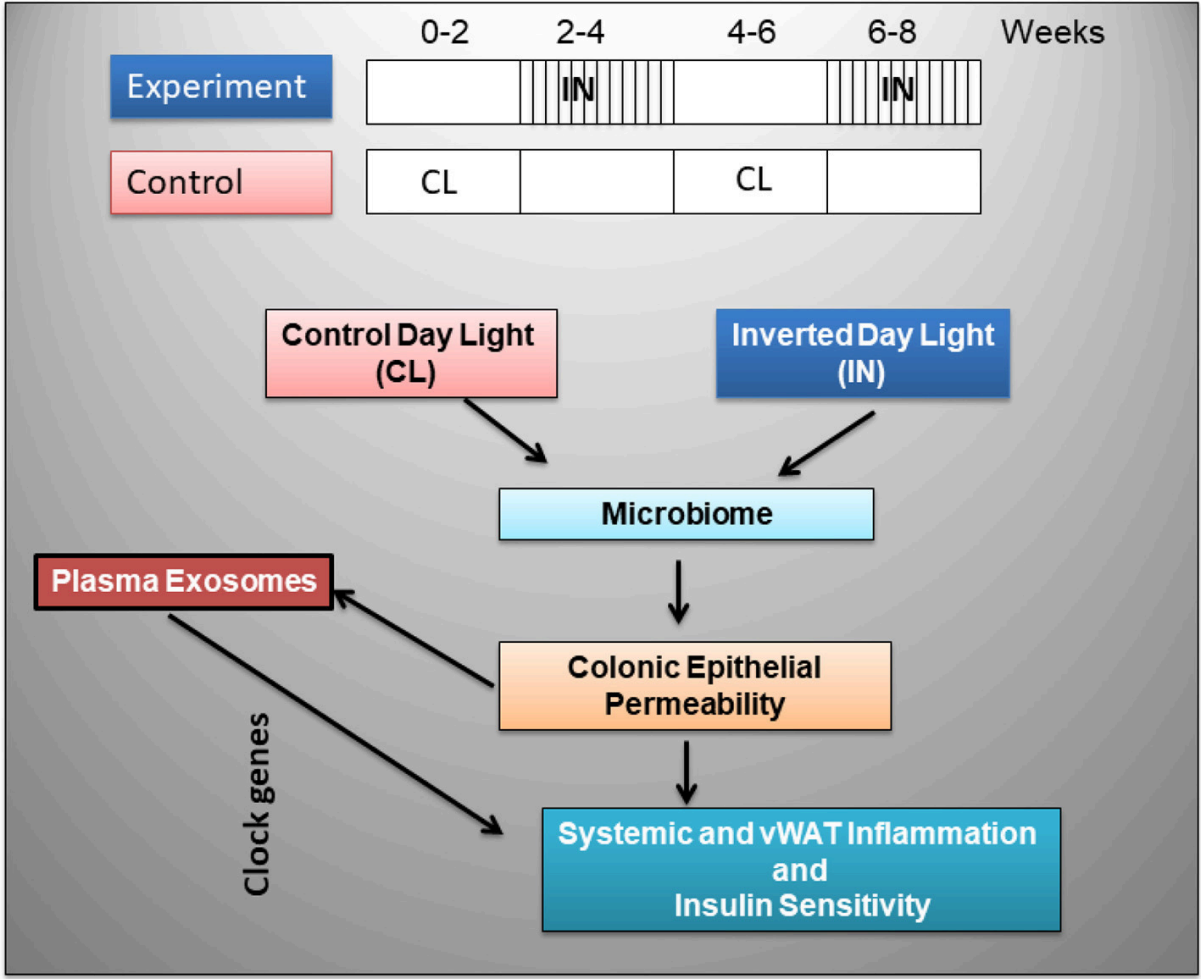
Schematic diagram illustrating the experimental design in mice exposed to inverted day light (IN) and control day light (CL) every 2 weeks for 8 weeks.

Strong evidence has revealed the presence of reciprocal interactions between the circadian system and organismal metabolism, which explains how disruption of body clocks by night shift work, travel induced jetlag, or frequent consumption of calorie-dense foods can all promote the occurrence of detrimental metabolic changes that contribute to obesity (Laermans and Depoortere, 2016). In addition, the gut microbiome has been shown to significantly contribute to the phenotypic characteristics of host, immunity, metabolism, and circadian clock function (Turnbaugh et al., 2007; Velagapudi et al., 2010; Leone et al., 2015; Lai et al., 2017), and sleep alterations have recently been implicated in altered gut microbiota promoting metabolic dysfunction (Leone et al., 2015; Poroyko et al., 2016). Thus, the study of novel circulating markers as potential candidates would emerge as a promising venue, and among those one of the widely and actively explored includes extracellular vesicles (EVs). Exosomes belong to a group of naturally secreted EVs mediating short- and long-distance intercellular communication delivering different kinds of biologically active cargo to recipient cells (Colombo et al., 2014; Maas et al., 2017). Such exosome-mediated cellular interactions may re-encode genes of target cells and play a part in cellular adaptive or injury mechanisms (Pisitkun et al., 2004; Gallo et al., 2012; Raposo and Stoorvogel, 2013; Khalyfa et al., 2016b,c,d,e). Therefore, we hypothesized that the sleep perturbations that characterize night shift work may be linked to obesity and to altered metabolic phenotypes via changes in circulating exosomes (Figure 1). Here we explored the effects of inverted light (IN) cycles mimicking night shift work schedules on metabolic function and gut microbiota properties on colonic epithelium permeability, and also investigated whether exosome functional properties would be altered and adversely affect naïve adipocyte cell substrate properties.

## Materials and methods

### Animals and light shift schedules

Male mice C57BL/6J (20–22 g) were purchased from Jackson Laboratories (Bar Harbor, ME, USA) and housed at a constant temperature (24 ± 1°C). Mice were fed normal chow diet, were housed in groups of 4–5 in a standard clear polycarbonate cages, and were allowed access to food and water *ad libitum*. Mice were randomly assigned to either control day light conditions (CL) or inverted light (IN) schedules consisting of alternation of light-dark cycles every 2 weeks for a total of 8 weeks (Figure 1). Animal cages were randomly assigned to IN (*n* = 15) or CL conditions (*n* = 15) groups. All efforts were made to minimize animal suffering and to reduce the number of animals used. Animal experiments were performed according to protocols approved by the IACUC of the University of Chicago (#72043).

### Body weights and blood tests

Body weight was measured weekly. At the end of the experiments, mice were fasted for 3 h, and blood samples were collected in vacutainer tubes containing EDTA (Becton Dickinson, Franklin Lakes, NJ). Blood was immediately centrifuged at 2,000 × g for 20 min at 4°C; subsequently, plasma samples were centrifuged for 5 min at 14,500 × g to remove remaining cells and platelets and immediately frozen at −80°C until further analyses.

Visceral fat was surgically removed, weighted, and a portion was immediately processed while the other was frozen in liquid nitrogen for further experiments. Blood glucose was measured using OneTouch Ultra2 glucometer (LifeScan, Milpitas, CA). Plasma insulin assays were performed with commercial ELISA kits (EMD Millipore, Billerica, MA) according to the manufacturer's protocol. Systemic insulin sensitivity was then assessed using the homeostasis model assessment of insulin resistance (Matthews et al., 1985).

### 16S rRNA sequencing of fecal samples

Inverted sleep cycle was introduced as follows, at day 15 of the protocol, the dark cycle was extended for 12 h, and the inverted light/dark cycle was maintained for 14 days. The light/dark cycle inversion was then reversed at day 28, and subsequently reinstated at day 43 for an additional 2 weeks (Figure 1). Fecal samples were collected at the end of each 2-week cycle, and DNA was isolated using Stool Fast Mini Kit (Qiagen, Valencia, CA). The 16S rRNA tag libraries were created using the set of indexed primers (V4 hypervariable region) and sequenced on an Illumina MiSeq platform (Caporaso et al., 2012). The unidirectional reverse sequences (mean length 150 bp) were also collected, processed and further annotated vs. RDP database (v.9) (Cole et al., 2014) using Mothur software (Schloss et al., 2009) for Phylotype-based analysis as described (Schloss et al., 2011; Kozich et al., 2013). Sequence clustering (at >97% identity) for identification of operational taxonomical units (OTUs), removal of chimeric sequences, and for generation of a read count table (i.e., tabulating the occurrence of each OUT in each sample) were performed with the software package. Metastats software (White et al., 2009) was used to determine differentially abundant taxa; PCoA and ANOSIM, Primers 7 (Clarke and Gorley, 2006), were used to compare groups and time points, the OTUs exhibiting differential dynamics were determined by ANOVA comparison of fitted models (for IN and Control groups) in R, ratio of fitted values IN vs. Control groups was calculated for days 7, 21, 35, and 50.

### Plasma exosome isolation and quantification

Exosomes were isolated from frozen plasma using the Total Exosome Isolation Kit according to the manufacturer's protocol (Life Technologies, Grand Island, NY, USA). Briefly, plasma samples were centrifuged at 2,000 × g for 20 min to remove cell/debris. The supernatants were precipitated by 0.2 volume of the Total Exosome Isolation Reagent (volume/volume). The mixtures were then incubated at 4°C for 45 min followed by centrifugation at 10,000 × g for 5 min. The supernatants were aspirated and removed, and the exosome pellets were then re-suspended in 1 × PBS buffer and subsequently stored at −80°C until used (Almendros et al., 2016; Khalyfa et al., 2016a). In a subset of preliminary experiments, the size of the isolated exosomes was evaluated using electron microscopy-based measurements.

The number of exosomes derived from CL or IN samples was determined for each sample using the Exocet kit (System Biosciences), according to the manufacturer's recommended protocols. The exosomes were lysed using a gentle lysis buffer, such as to maintain the enzymatic activity of the exosomal acetylcholinesterase (AChE) enzyme. A standard curve was then performed using known numbers of exosomes (as measured by NanoSight) and calibrated with a recombinant AChE enzyme standard solution provided in the kit. The average number of exosomes derived from CL samples were 6.0 × 10^7^/ml cells, while IN were 6.4 × 10^7^/ml. Equal numbers of exosomes were used for each experimental condition.

### Electric cell-substrate impedance sensing (ECIS)

Fresh fecal samples were obtained from CL and IN mice at the end of the experimental paradigm. Fecal samples were prepared in PBS to final concentration of 1 mg/ml (w/v), cleared by centrifugation (1 min, 5,000 rpm), filtered using 20 μm membrane filter (Millipore, Bedford, MA) and applied 15 μl per well, 10% (v/v) to ECIS system (Applied Biophysics, Troy, NY) to determine change of electrical resistance across monolayers of murine colonic epithelial cells (CACO-2; ATCC, Manassas VA), as previously described (Poroyko et al., 2016). Trans-monolayer capacitance of the monolayer was monitored continuously at high frequency (40 kHz) and the resistance at a low frequency (400 Hz). Experiments for each condition were always conducted in duplicate wells.

### Plasma measurements and limulus amebocyte lysate (LAL) activity

Plasma insulin was measured using commercially available enzyme-linked immunosorbent assay kits (Millipore, Billerica, MA). The appropriate range of the insulin assay was 0.2–10 ng ml^−1^, with the limit of sensitivity at 0.2 ng ml^−1^, and intra- and inter-assay variations at 3.73 and 10.52%, respectively, within the assay range. Lipid profiles including total cholesterol and triglycerides were determined using Infinity kits (Thermo Scientific, Pittsburgh, PA). Plasma samples were also used to determine the quantitative endpoint assay for the detection of gram-negative bacterial endotoxins. Bacterial endotoxin catalyzes the activation of a proenzyme in the modified Limulus Amebocyte Lysate (LAL) and photometrically measured at 405–410 nm. LAL assay was performed using Pierce LAL Chromogenic Endotoxin Quantitation Kit (#88282, Rockford, lL) according the manufacturer's protocol.

### Visceral fat and flow cytometry analysis

Epididymal fat pads corresponding to vWAT harvested samples were minced in KRB that was supplemented with 1% BSA and further incubated with collagenase (1 mg/mL; Worthington Biochemical Corporation, Lakewood, NJ) at 37°C for 45 min with shaking. Cell suspensions were then filtered through a 100 μm mesh, and centrifuged at 500 × g for 5 min to separate floating adipocytes from the stromal-vascular fraction (SVF) pellet. Individual SVF pellets were then re-suspended in FACS buffer (PBS plus 2% FBS) and 1 × 10^6^ cells were stained with fluorescence conjugate-primary antibodies or control IgGs at 4°C for 30 min. Cells were then washed twice and analyzed with a flow cytometer (Canto II; BD Biosciences, San Jose, CA), and data analysis was performed using the FlowJo software (Tree Star, Ashland, OR). Adipose tissue macrophages were defined as F4/80+ and CD11b+ cells, from which M1 and M2 macrophages were identified as CD11c+ or CD206+ cells, respectively, Tregs as Forkhead box P3+ (FoxP3+), and activated pro-inflammatory macrophages derived from bone marrow as Ly6c^high+^. All antibodies were from Biolegend (San Diego, CA). Specifically for FoxP3+, cells were permeabilized as described in the BioLegend's Cell Surface Immunofluorescence Staining Protocol. Briefly, 1 ml of 1X BioLegend's FOXP3 Fix/Perm solution was added to each tube, vortexed and incubated at room temperature in the dark for 20 min, cells were then spun down at 250 × g for 5 min, and the pellets were washed with 1 ml 1X BioLegend's FOXP3 Perm buffer. The cells were then re-suspended cells in 1 ml 1X BioLegend's FOXP3 Perm buffer, and incubated at room temperature in the dark for 15 min, followed by spinning down cells and discarding the supernatant. The pellets were resuspended in 100 μl of 1X BioLegend's FOXP3 Perm buffer, and 100 μl of flurochrome-conjugated anti-FOXP3 antibody was added and incubated at 4°C in the dark for 5 h. The cells were washed twice with staining buffer, and re-suspended in 0.3 ml cell staining buffer, and then analyzed with flow cytometry.

### Western blots

3T3-L1 (ATCC, Manassas, VA) cells were cultured in DMEM supplemented with 10% FBS and penicillin/streptomycin solution maintained in a humidified incubator at 37°C and 5% CO2 in 24-well plates. After reaching confluence, cells were incubated in differentiation medium containing dexamethasone (2.5 μM), 3-isobutyl-1-methylxanthine (0.5 mM), and insulin (10 μg/mL). After 4 days, the differentiation medium was replaced with the DMEM medium supplemented with 2% FBS and the cells were incubated with plasma exosomes isolated from IN or CL for 24 h. Adipocytes were then treated with 0 or 5 nm insulin (Sigma) at 37°C for 30 min prior to lysis. Serum was removed, and cells were washed with PBS. Cells were lysed in 2% SDS. The supernatants were collected after centrifugation at 15,000 × g for 15 min at 4°C. Protein concentrations of the cell lysates were determined using the BCA Kit (Life Technologies, Grand Island, NY). The lysates were subsequently separated on 12% SDS-acrylamide gel and transferred to nitrocellulose membranes (Millipore, Billerica, MA). After transfer, membranes were incubated in blocking buffer (5% non-fat dry milk in TBST) for 1 h at room temperature. The membranes were then incubated with phosphorylated Akt (Ser473) antibody (Cell Signaling Technology, Danvers, MA, USA) or with total Akt antibody (Cell Signaling Technology) overnight at 4°C. Membranes were then washed three times for 10 min each with 25 mm Tris, pH 7.4, 3.0 mm KCl, 140 mm NaCl and 0.05% Tween-20 (TBST), incubated with anti-rabbit immunoglobulin G:HRP-linked antibody (Cell Signaling Technology) in blocking buffer with gentle agitation for 1 h at room temperature. Immunoreactive bands were visualized using an enhanced chemiluminescence detection system (Chemidoc XRS+; Bio-Rad, Hercules, CA), and quantified by the Image Lab software (Bio-Rad, Hercules, CA).

### Quantitative reverse transcription-polymerase chain reaction (qRT-PCR)

Total RNA was isolated from 3T3-L1 cells (total *n* = 8/experimental group), cells treated with CL- or IN-derived exosomes, using The RNeasy Lipid Tissue Mini Kit following the manufacturer's instructions (Qiagen). Briefly, 3T3-L1 cells were maintained in growth medium with 10% fetal bovine serum (FBS; Clonetics) and incubated at 37°C and 5% CO_2_ in cell culture incubator until confluence. Cells were washed with DMEM media containing 2% FBS and exosomes were added for 24 h. The RNA quality and integrity were determined using the Eukaryote Total RNA Nano 6000 LabChip assay (Agilent Technologies, Santa Clara, CA) on an Agilent 2100 Bioanalyzer. qRT-PCR gene expression assays (TaqMan; Applied Biosystems, Foster City, CA) were performed using TaqMan gene expression. The clock genes included: Aryl Hydrocarbon Receptor Nuclear Translocator Like (*ARNTL, BMAL1*), Period (*PER1*), and Cryptochrome (*CRY2*) were assessed by qRT-PCR using high-capacity RNA to complementary DNA (cDNA) Master Mix (Applied Biosystems) and TaqMan PCR Master Mix (Applied Biosystems). Reverse transcription of total RNA was conducted with TaqMan reverse transcription reagents RT-PCR kit according to the manufacturer's instructions (Applied Biosystems). qRT-PCR was performed for mRNAs in triplicate in ABI PRISM 7500 System (Applied Biosystems, Foster City, CA). Ribosomal 18S rRNA was used as a reference gene to normalize the expression ratios. The mean cycle number (Ct) values of the 18S Ct and the gene of interest Ct were calculated. The relative expression of the gene of interest was calculated using the 2−ΔΔCt method.

### Statistical analysis

All data are reported as mean ± standard error. Comparisons of all quantitative data groups were performed using unpaired Student *t*-tests. For all comparisons, a *P* < 0.05 was considered as statistically significant.

## Results

### Body weight and metabolic parameters

Mice exposed to IN had higher body weight compared to CL mice (Table 1; *P* < 0.03). Of note, IN mice had significantly increased visceral fat weight vs. CL mice (*P* < 0.04; Table 1). Total cholesterol levels were significantly higher in IN mice (*P* < 0.03), and triglyceride levels were also elevated (*P* < 0.001) compared with CL (Table 1). HOMA-IR index was calculated to evaluate the impact of shiftwork-like schedules on a surrogate indicator of systemic insulin sensitivity. IN mice exhibited increases in plasma glucose and HOMA-IR values were significantly increased compared with CL mice (*P* < 0.02). Taken together, these data indicate that IN is associated with systemic insulin resistance and dislipidemia in mice.

**Table 1:** Body weight, vWAT mass, metabolic parameters, and Limulus Amebocyte Lysate (LAL) detection assay in IN and CL exposed mice.

	**SC**	**IN**	***p*-value**
Body weight (g)	27.29 ± 1.28	28.5 ± 1.05	<0.03
vWAT (mg)	374.08 ± 8.11	417.38 ± 37.06	<0.04
Cholesterol (mg dl−1)	83 ± 4.31	111 ± 5.61	<0.04
Triglycerides (mg dl−1)	81.4 ± 11.71	92.23 ± 5.26	<0.001
HOMA-IR	1.91 ± 0.63	3.22 ± 0.52	<0.02
LAL activity (EU/ml)	0.41 ± 0.05	0.62 ± 0.08	<0.02

*vWAT, visceral white adipose tissue; Limulus Amebocyte Lysate (LAL) is a Chromogenic Endotoxin Quantitation Kit for the detection of gram-negative bacterial endotoxins*.

### Fecal microbiota

**Figure 2:**
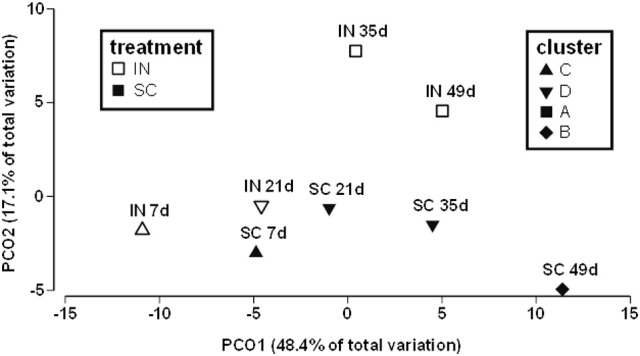
Gut microbial community is altered by IN compared to CL. Principal Coordinate analysis (PCoA) plot depicts structural changes in microbial communities over the period of 8 weeks, and the results of k-mean clustering (*n* = 4).

The fecal material is widely accepted as a proxy for intestinal microbiota studies (Turnbaugh et al., 2007, 2009; Raoult and Henrissat, 2014; Thomas et al., 2015; Yasuda et al., 2015). The composition of gut microbes in stools in IN and CL mice were evaluated using 16S rDNA sequencing, with the composition of bacteria and bacterial fecal being profiled at the levels of phylum, class, order, family, genus, and species (Table 2 and Figure 2). The PCR amplicons were sequenced, rarefied to 10,000 sequences/sample, taxonomically annotated and assembled in 82 OTUs according to the lowest (genera) level of annotation. Median OTU size is 36 sequences. To assess the effect of IN, the table of OTU abundances was used to calculate distances between samples using Bray-Curtis dissimilarity measure (Legendre et al., 1998). OTU abundances were, standardized, square root transformed and used to calculate dissimilarity matrix. The matrix was further used to calculate distances between group centroids [time (w) of treatment (IN or control)] and visualize distribution of group centroids using Principal Coordinate analysis (PCoA) plot (Figure 2), demonstrating that IN treatment accounts for 17.1% of total dataset variation. The difference of gut microbiota between IN and control group became apparent at day 35 as demonstrated by k-clustering analysis, and persisted thereafter (Figure 2).

**Table 2:** List of significantly operational taxonomic units (OTUs) affected in inverted light compared to sleep control.

OTUs^*^	**Phylum**	**Family**	**Genus**	**Fitted Ratio IN/CL (Day 49)**	**ANOVA (*p*-value)**
10	Bacteroidetes	*Porphyromonadaceae*	*Barnesiella*	2.29	0.0002
16	Firmicutes	*Ruminococcaceae*	*Clostridium_IV*	1.47	0.0008
17	Firmicutes	*Erysipelotrichaceae*	*Turicibacter*	0.58	0.002
18	Firmicutes	*Lachnospiraceae*	*Clostridium_XlVb*	3.16	3.16E-06
19	Firmicutes	*Lactobacillaceae*	*Lactobacillus*	9.6	0.001
22	Firmicutes	*Ruminococcaceae*	*Pseudoflavonifractor*	3.44	8.52E-06

* *OTUs unambiguously classified to the genus level were selected. The OTU abundances were used to calculate distances between samples using Bray–Curtis dissimilarity measure*.

The dynamics of individual genera, with abundance above 1%, was analyzed using ANOVA corrected for multiple testing. Genera with *P* < 0.03 were considered exhibiting dynamics significantly different from control. The total of 10 OTUs of genus level were selected, of them, the majority (7 OUTs) belong to phylum *Firmicutes*, 2 OTUs were attributed to phylum *Bacteroidetes*, and 1 OUT was identified as “unclassified Bacteria.” Table 2 illustrates the subset of 6 OTUs, unambiguously classified to the level of genus, and demonstrates that the IN paradigm is associated with the increase of abundance of 5 genera *Clostridium XIVb, Clostridium IV, Pseudoflavonifactor, Barnesiella*, and *Lactobacillus*. Genus *Turicibacter, was* elevated at day 21 and 35, but at day 50, it exhibited 0.5-fold decrease relative to CL.

The LAL assay revealed significant increases in LAL (EU/ml) for IN (0.62 ± 0.08; *n* = 15) vs. CL (0.41 ± 0.05; *P* < 0.017; *n* = 15).

### Effect of stool fecal water samples on colonic epithelium barrier disruption

**Figure 3:**
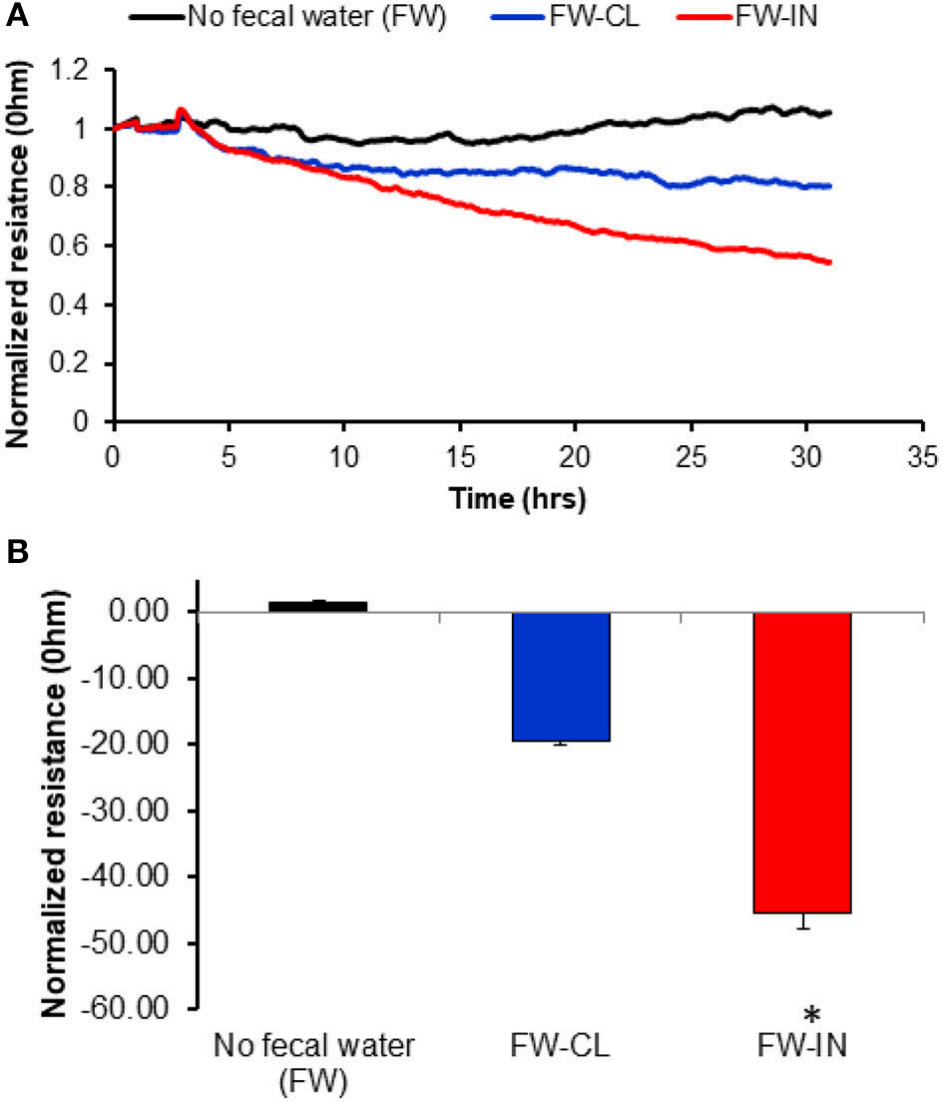
Effects of fecal water derived from IN mice upon completion of the exposures on colonic epithelial cell monolayer resistance using electric cell-substrate impedance sensing (ECIS). Panel **(A)** shows the average tracings over time of normalized resistance across a monolayer of Human Normal Colon Cells (HNCC). Panel **(B)** Changes after 24 h of exposure (^*^P < 0.0003). Data are presented as mean ± *SD*; *n* = 15/experimental condition.

To study the effects of fecal water on colonic epithelium barrier disruption *in vitro*, we used ECIS measurements in monolayers of HNCC exposed to IN and CL “fecal water” as demonstrated by mean TER changes. Normalized resistance tracings in HNCC cell monolayers (Figure 3) showed rapid decreases in trans-monolayer resistance induced by contact with “fecal water” samples from IN, but not from CL mice (*p* < 0.001; *n* = 15/experimental group). Of note, there was a significant association between LAL assay levels and the degree of monolayer resistance decreases (*r*:0.63; *p* < 0.001).

### Effect of plasma-derived exosomes on mouse adipocyte (3T3-L1) cell and insulin sensitivity

**Figure 4:**
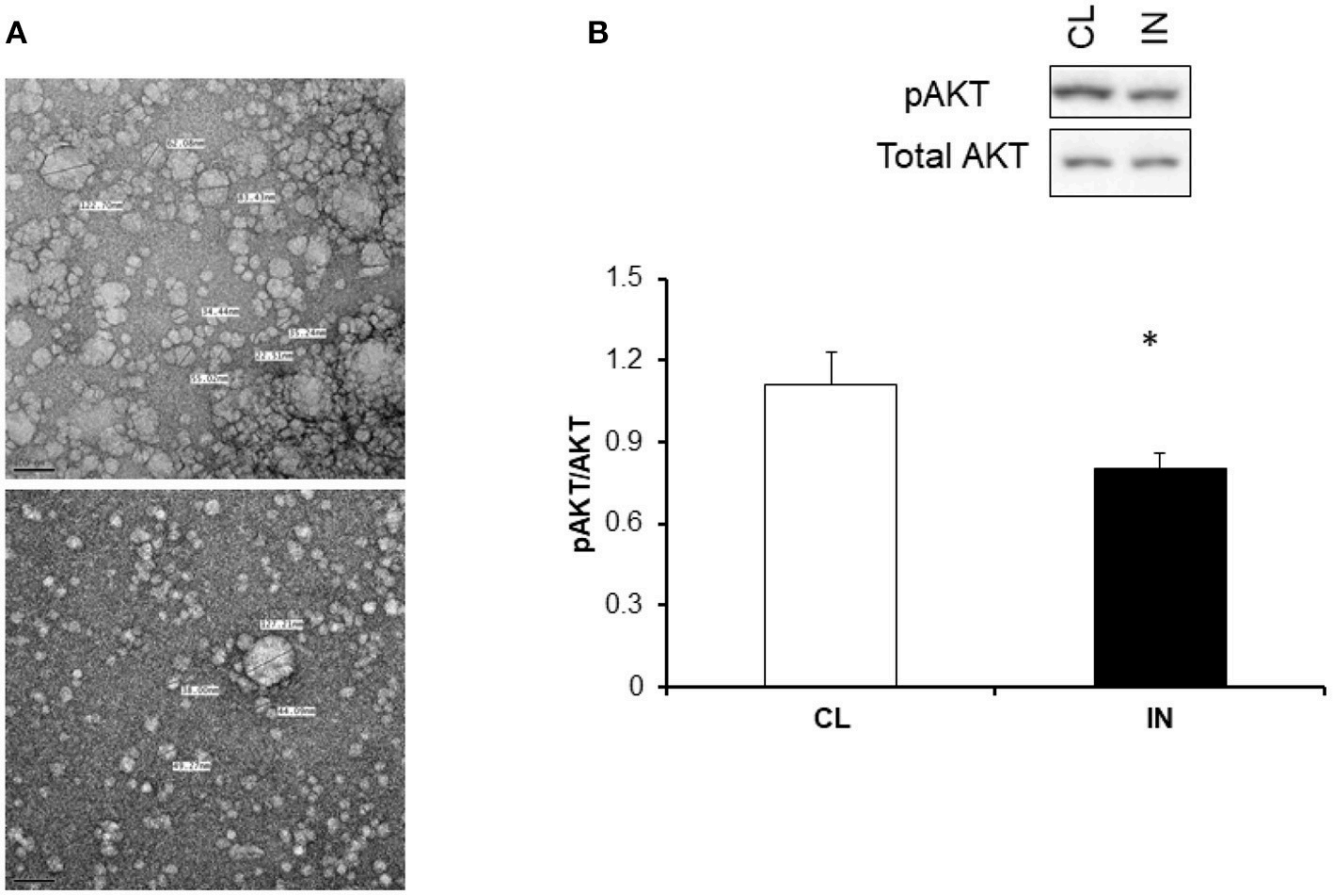
**(A)** Representative electron microscope images and associated microvesivcle measurements illustrating the correct and predominant size corresponding to exosomes (30–100 nm). **(B)** Effects of plasma exosomes from IN and CL mice on naïve mouse adipocytes. Exosomes were added into differentiated adipocytes 3T3-L1 cell culture for 24 h and followed by treatment with 5 or 0 μm of insulin and then examined for phosphorylated AKT and total AKT. A significant reduction of pAKT/AKT after exogenous insulin in 3T3-L1 treated with IN exosomes emerged compared to CL (*P* < 0.001; *N* = 15/group, ^*^Indicates statistical significance).

To evaluate the *in vitro* effects of plasma exosomes derived from IN or CL on insulin sensitivity, differentiated adipocytes cells (3T3-L1) were treated with exosomes for 24 h with insulin and without insulin for 30 min and Akt phosphorylation was appraised using western blots. The increases in the expression of phosphorylated AKT (pAKT) relative to total AKT expression elicited by insulin treatment were significantly reduced in IN mice compared to CL mice (Figure 4).

### vWAT macrophage populations

**Figure 5:**
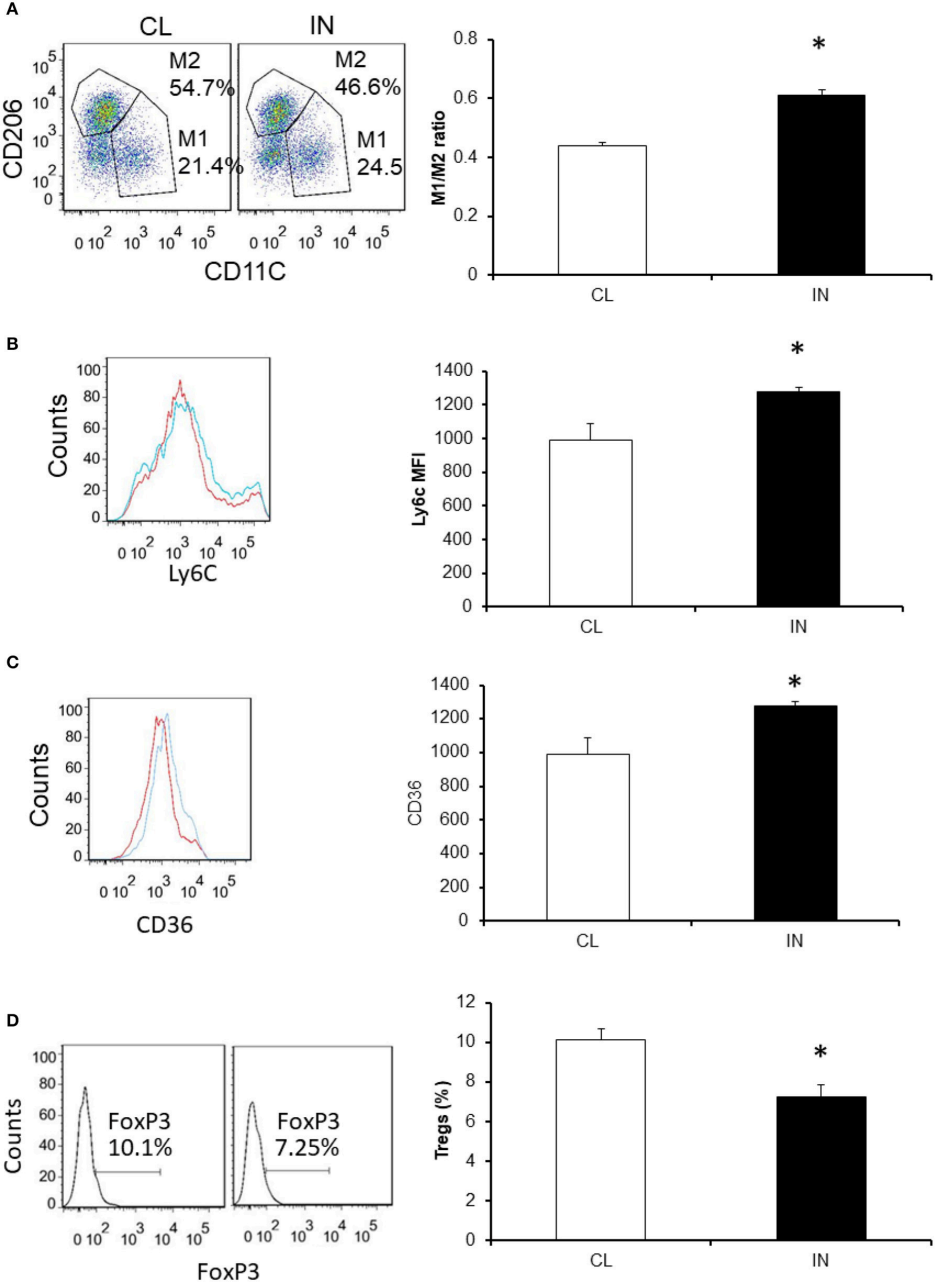
Effects of IN exposures on vWAT macrophage and Treg populations using flow cytometry. Panel **(A)** is a representative example for CD11C^+^ and CD206^+^ that were used for double positive for macrophages. M1 and M2 macrophages were also identified as CD11c^+^ or CD206+ cells, respectively. Panel **(B)** is a representative example for resident macrophages (CD64). Panel **(C)** is a representative example for M1-like metabolic macrophages (CD36hi ++) in vWAT. Blue color is for SC and red color for IN. Panel **(D)** is a representative example for Tregs (FoxP3 + and CD4+ cells) in vWAT. Data are shown as means ± *SD* (*n* = 8/experimental group). ^*^Indicates statistical significance; *p* < 0.01.

To examine the potential impact of IN on vWAT inflammation, we evaluated changes in macrophages and Treg lymphocytes (Figure 5). We found that mice exposed to IN exhibit increased total numbers of macrophages in the VWAT compared with CL (Figure 5; *n* = 8/experimental group; *P* < 0.03). We also found the M1/M2 ratios were significantly increased in IN vs. CL (0.44 ± 0.01 vs. 0.61 ± 0.02; *P* < 0.03). Increases in Ly6c^high+^ cells emerged in IN mice suggesting a rise in pro-inflammatory macrophage recruitment from the bone marrow to the vWAT, and such changes were absent in CL mice (*P* < 0.05; Figures 5A,B). In addition, scavenger receptor CD36 expression, which reflects metabolic dysfunction in vWAT (Kratz et al., 2014), was increased in the VWAT of IN mice (mean fluorescence intensity: 895 ± 65 vs. 724 ± 38; *P* < 0.03), as was the percentage of CD36+ macrophages. We also found that IN mice showed significant decreases in FoxP3+ cells as compared to CL mice, suggesting a decrease in vWAT Treg lymphocyte population (*P* < 0.04; Figures 5C,D).

### Effects of exosomes derived from inverted day light on clock gene expressions

**Figure 6:**
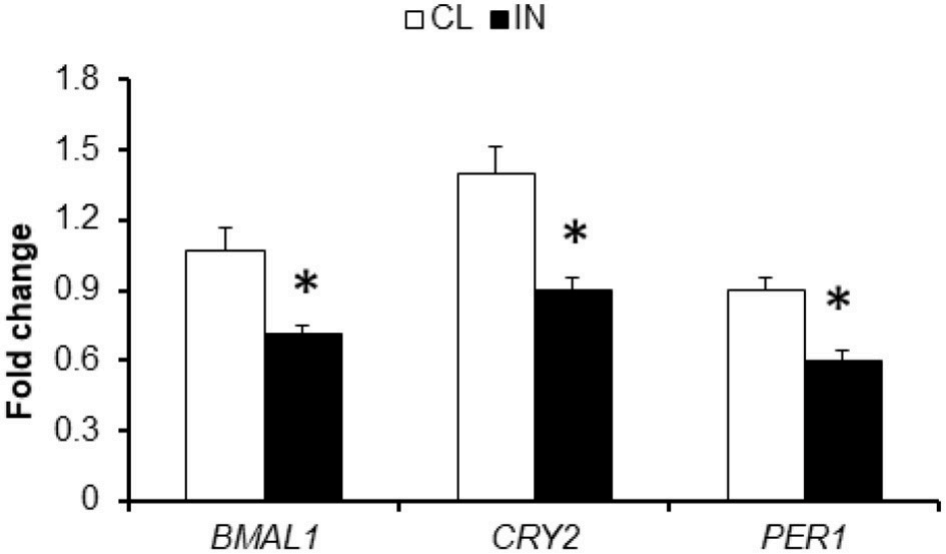
Plasma exosomes from mice exposed to IN alter clock gene expression in vWAT. Total RNA was isolated from vWAT and analyzed by qRT-PCR analysis for clock genes mRNA expression. qPCR data were normalized to 18 s rRNA as an internal control. *BMAL1, CRY2*, and *PER1* expression were decreased by exosomes from IN mice compared to CL mice. Data are presented as mean ± *SD*; *n* = 8/experimental condition; ^*^Indicates statistical significance *P* < 0.01.

We next studied the effects of exosomes on clock gene expression in naïve adipocytes using exosomes derived from IN and CL mice. Data were obtained by qRT-PCR for three canonical clock genes, namely *BMAL1, CRY2*, and *PER*1 (Figure 6). We found IN-derived plasma exosomes (*n* = 6/experimental group) led to reduced expression of *BMAL1* (*P* < 0.002), *CRY2* (*P* < 0.006), and *PER1* (*P* < 0.03, Figure 6).

## Discussion

Epidemiologic evidence shows that shift and night work are associated with elevated risk of metabolic disorders, perhaps in part as a result of chronic circadian misalignment and poor physiological adaptation to sleep loss, both leading to chronic sleepiness and feeding at unfavorable circadian times (Scheer et al., 2009; Hansen et al., 2016; Proper et al., 2016; Wirth et al., 2017). Sleep deficits and circadian disruption are strongly associated with metabolic dysregulation and may contribute to weight gain, obesity, and type 2 diabetes, potentially by altering timing and amount of food intake, disrupting energy balance, inflammation, impairing glucose tolerance, and insulin sensitivity (Ye et al., 2013; Kawabe et al., 2014). In the present study, we show that altered prolonged circadian light exposures mimicking nighttime shiftwork in mice are associated with a constellation of temporal changes in fecal microbiota, increased colonic epithelial permeability and plasma endotoxin levels, pro-inflammatory modifications in vWAT macrophages, Tregs, and increased systemic HOMA-IR, indicative of insulin resistance. Furthermore, circulating exosomes from IN-exposed mice elicit *in vitro* a reduction of pAKT/AkT ratio responses to exogenous insulin in naïve adipocytes indicating insulin resistance while also altering expression of clock genes. Taken together, these findings suggest that the functional effects of circulating exosomes released under IN conditions *in vitro* may be indicative of selective cargo changes that play a mechanistic role in the increased risk for the metabolic dysfunction frequently found in shift workers.

Shift work including a nighttime rotation is common, with up to 20% of the Western workforce encountering alternate work schedules (Kivimaki et al., 2011). The most common health complaint of shift workers is lack of sleep (Lu et al., 2017b), association with cardiovascular disease (Morris et al., 2016; Lu et al., 2017a), and gastrointestinal disorders (Koh et al., 2014). Several lines of evidence including human epidemiology and animal models indicate that disruption of a regular 24-h light-dark cycle increases morbidity and mortality (Blask et al., 2005; Stevens et al., 2007). In fact, our body is unprepared for night-time calorie intake, and lipid profiles including has shown circadian rhythmicity, with higher levels at night than during the day (Holmback et al., 2003). It has been demonstrated that misalignment of circadian rhythms among night workers is associated with increased insulin resistance and altered plasma lipids (Al-Naimi et al., 2004). Our current findings show that in mice exposed to IN had higher insulin levels and HOMA-IR, as well as elevated triglycerides and cholesterol levels (Table 1), thereby recapitulating previous findings in humans (Karlsson et al., 2003; Sookoian et al., 2007). Thus, the IN paradigm emerges as a robust mimic of night shiftwork schedules.

Increasing evidence suggests that gut microbes may shape host metabolic and immune network activities, and ultimately influence the development of metabolic dysfunction including obesity and diabetes (Hsiao et al., 2013; Ridaura et al., 2013; de Groot et al., 2017; Wen and Duffy, 2017). The intestinal microbiota of both mice and humans displays diurnal oscillations that are influenced by feeding rhythms, thereby ensuring optimal compositional and functional profiles throughout the 24-h cycle (Thaiss et al., 2014). With the advances of 16S rRNA sequencing, information on how host-associated microorganisms influence host physiology, behavior, and health has begun to emerge (Gontang et al., 2017; Hu et al., 2017), whereby more specific roles for certain types of bacterial species in mediating host immunity and immunologic diseases is becoming apparent. In particular, the segmented filamentous bacteria have been found to promote autoimmune arthritis through an enhanced Th17 response (Ivanov et al., 2009). We show in Table 2 that the list of significant OTUs affected in IN is associated with functional effects on epithelial barrier as illustrated by trans-epithelial electric resistance measurements and LAL activity levels in plasma. We therefore propose that IN schedules induce shifts in gut microbiota that promote increased colonic permeability and lead to increases in bacterial translocation to the systemic circulation (i.e., elevated LPS), where they elicit pro-inflammatory changes in target organs such as vWAT, resulting in increased M1 macrophage and reduced Treg populations, ultimately manifesting as insulin resistance.

Gut microbiota play a vital role not only in the digestion and absorption of nutrients, but also in homeostatic maintenance of host immunity, metabolism and the gut barrier, and the alterations of gut microbiota can contribute to the pathogenesis of metabolic disorders such as obesity, diabetes mellitus and non-alcoholic fatty liver disease. Despite the fact that there is a causal link between gut microbiota and these metabolic disorders, the potential role of microbiota altering circulating exosomal cargo and operating as the intermediate mediators of the metabolic changes described in our study prompted us to explore the functional implications of plasma exosomes. Indeed, in two recent studies by the same investigative group, one related to the gut bacteria-derived extracellular vesicles (EVs) which can play an important role in maintenance of immune homeostasis in the gut (Kang et al., 2013), and the other related to gut microbe-derived EVs that might be key players in the development of insulin resistance and impairment of glucose metabolism (Choi et al., 2015), further enhanced the viability of our hypothesis. In the current study, we proposed that the effects of IN on the microbiota may lead to changes in the cargo of circulating exosomes, which in turn may affect the metabolic changes in adipose tissues. However, the study does not negate another possibility, namely that exosomes will be more directly modified by IN and resultant circadian clock misalignment, and that such exosomes will in turn induce functional changes in different organs, including gut and gut microbiota as well as adipose tissues.

Indeed, accumulation of macrophages and changes in their polarity in adipose tissues has been correlated with increasing body weight and insulin resistance (Xu et al., 2003). vWAT is known to play a central role in metabolic regulation, and low grade inflammation in this target organ is a key process in the emergence of insulin resistance. At the cellular level, macrophages, lymphocytes and adipocytes are known to interact and regulate the inflammatory cascade and metabolism (Shaul et al., 2010). In the metabolic syndrome and diabetes, the proportion of pro-inflammatory M1 polarized macrophages heavily outweighs that of M2 macrophages (Lumeng et al., 2007). Furthermore, M1 macrophages secrete pro-inflammatory cytokines including TNFα, IL-1β, and IL-6, some of which can directly alter insulin receptor signaling in adipocytes, leading to insulin resistance. In a rodent model, circadian disruption due to shifted light schedules induced increases in innate immune responses, and this circadian desynchronization resulted in enhanced TNF-α and IL-6 production after LPS stimulation (Guerrero-Vargas et al., 2015). In contrast, Tregs function to counteract the development of chronic inflammation in vWAT, are highly enriched in lean adipose tissue, and maintain insulin sensitivity by limiting inflammation, and producing insulin sensitizing cytokines, such as IL-10 (Winer et al., 2009). In addition to the unfavorable macrophage and Treg profiles in IN, we also found increased numbers of Lyc6c^high^ positive cells in the vWAT from IN mice compared to CL mice, which represent monocytes recruited from the bone marrow to the vWAT where they differentiate into macrophages and participate in vWAT inflammation (Yang et al., 2014).

A novel and exciting aspect of our study involved exploration of the effects of circulating exosomes on naïve adipocytes. We found that IN-derived exosomes can adversely impact insulin sensitivity of naïve adipocytes, as illustrated by exogenous insulin-induced Akt phosphorylation responses. Furthermore, these findings suggest that plasma exosomes may be contributing to the decreased insulin sensibility in IN mice compared to CL mice. Thus, circulating exosomes could represent promising biomarkers of the IN, and enable identification of at-risk subjects for altered metabolic developmental trajectories. Furthermore, we investigated the effects of exosomes derived from IN and CL on the expression of clock genes in naïve adipocytes. We found that IN exosomes alter *BMAL1, CRY2*, and *PER1* expression. At the molecular level, generation of oscillations by the circadian pacemaker depends on the concerted co-expression of a set of clock genes, including *CLOCK, BMAL1, PER1-3, CRY1-2*, and these genes participate in several intricate interlocked transcription-translation feedback loops, through which they not only regulate their own expression, but also that of numerous downstream clock-controlled genes (Laermans and Depoortere, 2016). The molecular circadian feedback loop regulates the expression of clock-controlled genes in a rhythmic manner, resulting in the oscillation of tissue-specific metabolic and physiological functions (Yoo et al., 2004). However, the mechanism through which such intercellular communication is effected are unclear (Takahashi, 2015; Ehlen et al., 2017; Paschos and FitzGerald, 2017). Our current findings suggest that exosomes may provide a vehicle to communicate to peripheral tissues the circadian clock misalignment introduced by IN, which ultimately disrupts adipocyte homeostasis resulting in altered metabolic function, as reflected by insulin resistance. It remains unclear whether exosomes derived from IN mice will also modify the function of other important metabolic cellular targets, such as hepatocytes, myocytes or pancreatic β cells.

Among the limitations of our study, we should point out that we did not specifically examine which of the fecal microbiota UOTs that are affected underlies the changes in colonic epithelium permeability and downstream systemic effects. Similarly, we did mot explore changes in exosome cargo that account for their effects on clock gene expression and insulin resistance. Although, such efforts are beyond the scope of the current work, it is probably best to directly assess them in humans, particularly considering that the majority of the functional outcomes reported herein reflect *in vitro* reporter assays.

This study is a proof of concept illustrating that gut microbiota and the cargo of plasma exosomes are altered by periodic shifts in environmental lighting, and that these changes are associated with concurrent changes in metabolic function. We further provide initial observations that microbiota alterations in IN may lead to altered exosomal cargo, which then induces adipose tissue insulin resistance, possibly via induction of systemic inflammation and altered clock expression in target tissues. Alternatively, IN could directly change exosome cargo, and then such cargo could functionally alter the gut microbiome and also adipose tissues. We did not conduct sleep studies on the animals since instrumentation of the animals could further modify and perturb the parameters we were keen on acquiring (gut microbiota, circulating exosomes). Despite the fact that there are clear limitations to the present study, there is also strong evidence derived from both animal and human experimental models to suggest that disrupted sleep and circadian misalignment can contribute to weight gain, obesity and adverse metabolic health (McHill and Wright, 2017). Furthermore, the interdependence of circadian physiology and sleep-wake cycle is not negated, but the latter was not explored. Indeed, imposed disturbances in the light/dark cycle, in sleep/wake schedules, or in feeding/activity behaviors can independently and reciprocally affect each other and also alter the circadian functioning of the clocks located in the brain and in the peripheral tissues, with these alterations having been associated with impaired glucose tolerance and type 2 diabetes (Vieira et al., 2014). A major challenge remains in understanding the interplay between brain and peripheral clocks, and in determining how these interactions promote energy homeostasis across the sleep-wake cycle under both physiological and pathological conditions. We suggest here that circulating exosomes, either directly or indirectly via the gut microbiome play a role in the exquisite physiological coordination (CL) or IN-induced misalignment between central and peripheral clocks.

## Conclusions

In summary, in a simple murine model of chronic nocturnal shift work, significant changes in gut microbiota and increased colonic cell permeability appear to induce not only systemic and vWAT inflammatory changes and insulin resistance, but also alter the cargo and function of plasma exosomes which appear to underlie components of such effects, possibly by communicating the circadian clock misalignment to target organs. Such findings open the possibility of identifying specific exosomes cargo constituents that are altered by periodic shifts in environmental lighting, and convey such circadian misalignment to target organs involved in metabolic homeostasis.

## Author contributions

AK performed experiments, analyzed data and drafted the initial version of the manuscript. VP, ZQ, AG, AAK, and MA performed experiments and analyzed data. IA and RF provided blinded assessments and critical input to manuscript drafts. DG provided the conceptual framework for the project, supervised experiments, analyzed data, and edited the manuscript. All authors have reviewed the final version of the manuscript and given their approval. DG serves as the guarantor of the paper, and takes responsibility for the integrity of the work as a whole, from inception to published article.

### Conflict of interest statement

The authors declare that the research was conducted in the absence of any commercial or financial relationships that could be construed as a potential conflict of interest.
